# Supplementary material for: Trypanosoma cruzi Isolates Naturally Adapted to Congenital Transmission Display a Unique Strategy of Transplacental Passage
Source: Microbiol Spectr. 2023 Feb 14;11(2):e02504-22. doi: 10.1128/spectrum.02504-22 (PMC10100920; doi:10.1128/spectrum.02504-22)

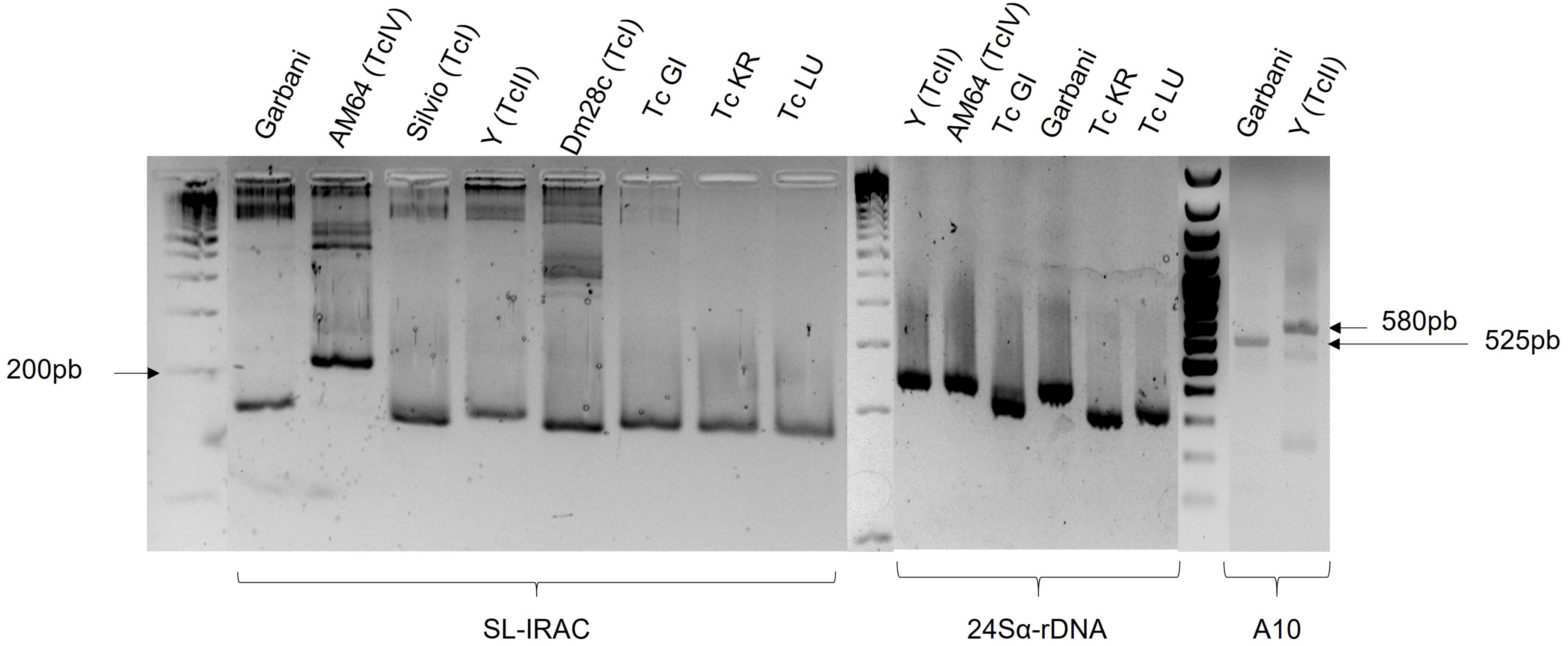

PCR fragment size obtained for the different strains

Strain	SL-IRAC	24Sα-rDNA	A10	DTU
Dm28c (Reference)	150	-	-	TcI
Y (Reference)	157	140	580	TcII
AM64 (Reference)	200	140	-	TcIV
Garbani	157	140	525	TcVI
Tc GI	157	125	-	TcV
Tc KR	157	125	-	TcV
Tc LU	157	125	-	TcV

Figure S1.
Molecular typification of the different *T. cruzi* strains by PCR method described by Cura et al., 2015

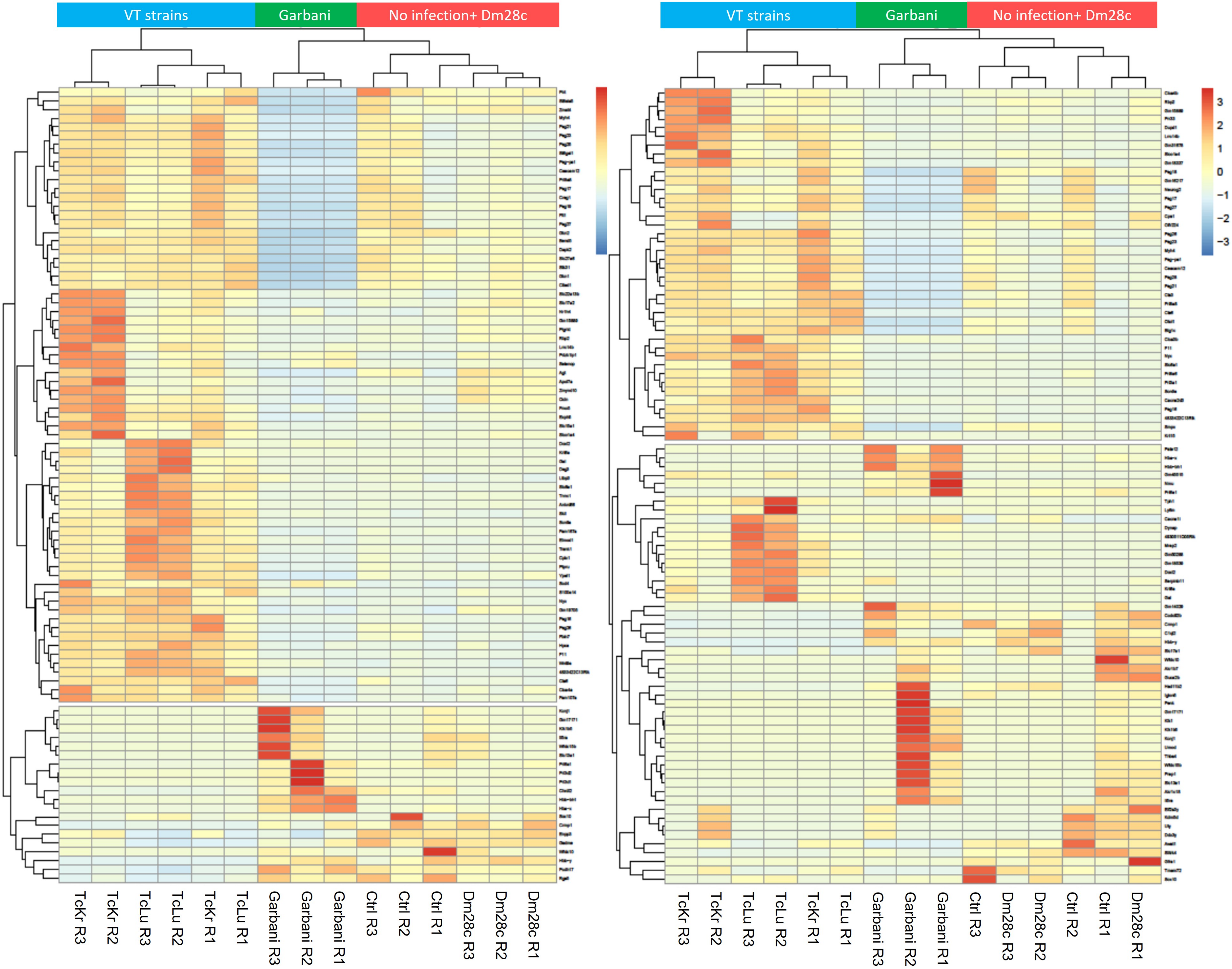

Figure S2. Cluster analysis showing normalized read counts for the top 30 more significative DEGs (left) and top 30 genes that change the most (right) in the different biological replicates within each experimental group.

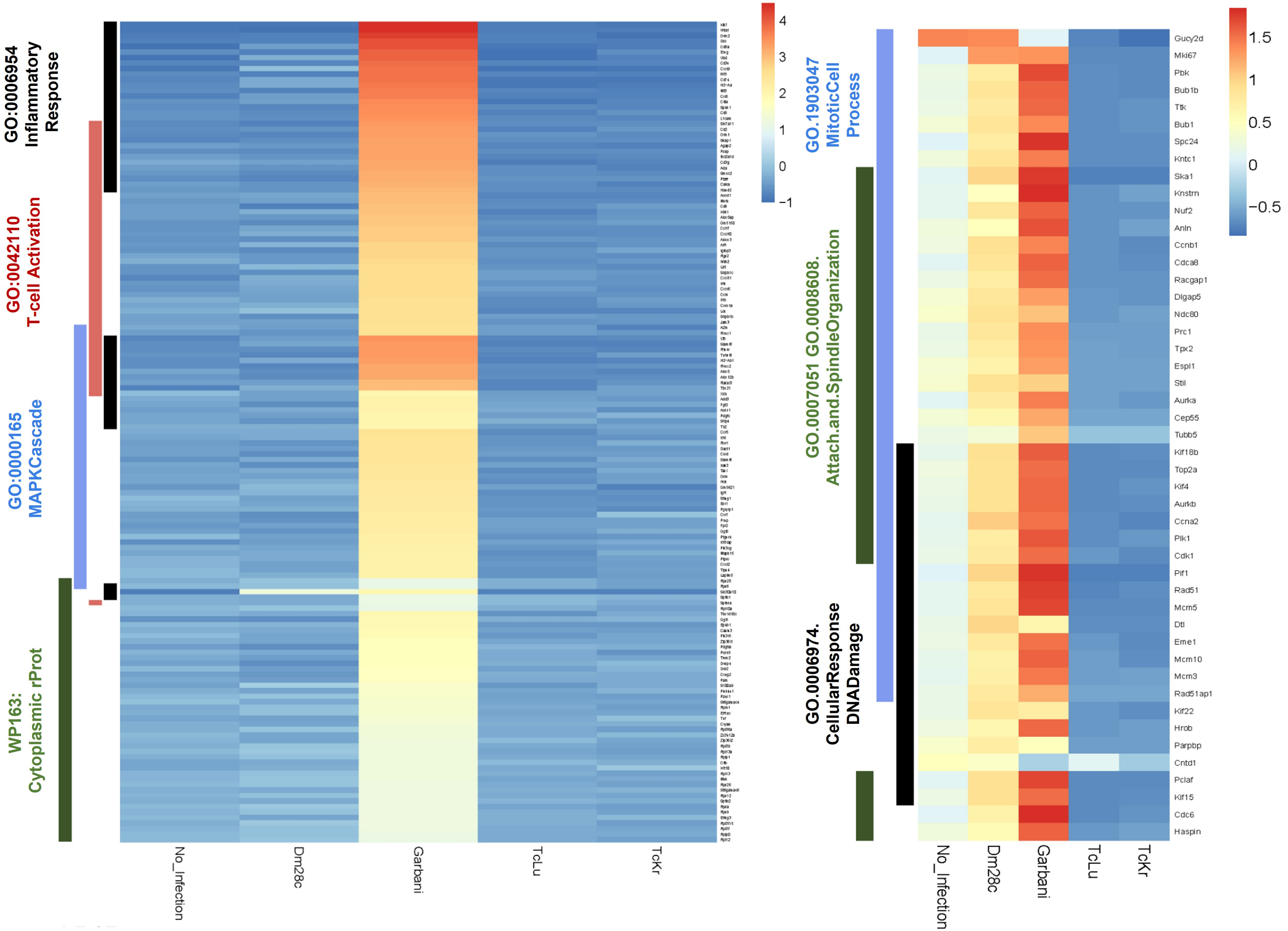

Figure S3. Heatmaps of total read counts of genes belonging to GO Terms or Pathways significantly up regulated in placentas infected with Garbani (left) and Go Terms significantly Down regulated in placentas infected with VT strains (right).

Supplementary figure 4.

Heatmaps represent row scaled normalized mean (biological replicates) read count values for each strain infected placenta. Each heatmap shows the behavior of all genes from a selected pathway.

1. **Immune response (blue box indicates genes related to tolerance and anti-inflammatory processes, upregulated in VT strains)**
2. **T-cell activation**
3. **Inflammatory response**
4. **Cell junctions**
5. **Cell cycle and other related**
6. **Mitotic cell cycle process**
7. **Cell projections and morphogenesis**
8. **Import to the cell**
9. **Positive regulation of secretion and import**
10. **Positive regulation of secretion**
11. **Ribosomal citoplasmatic proteins**

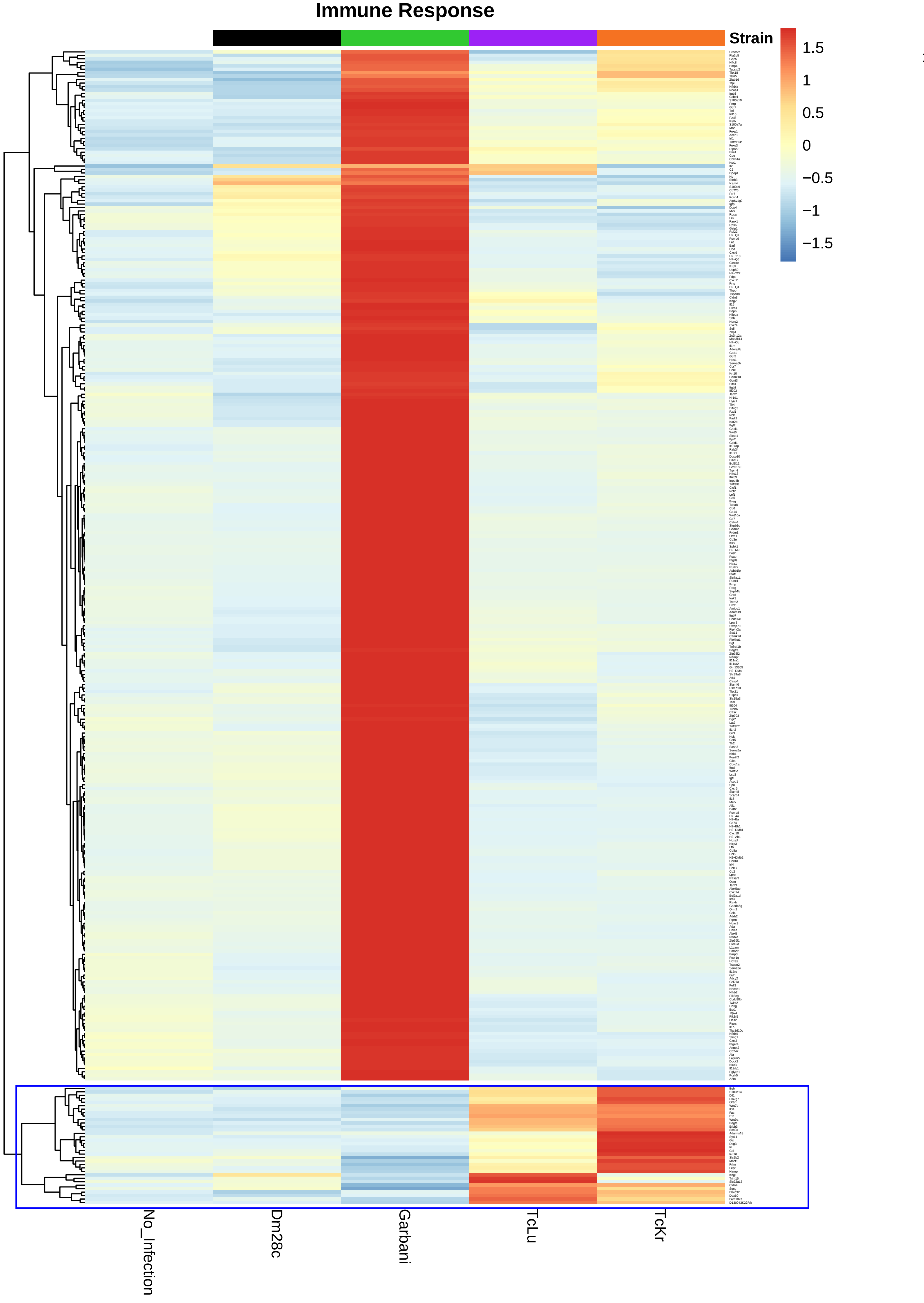
Immune Response
Strain
1.5
1
0.5
0
−0.5
−1
−1.5
No_Infection
Dm28c
Garbani
TcLu
TcKr

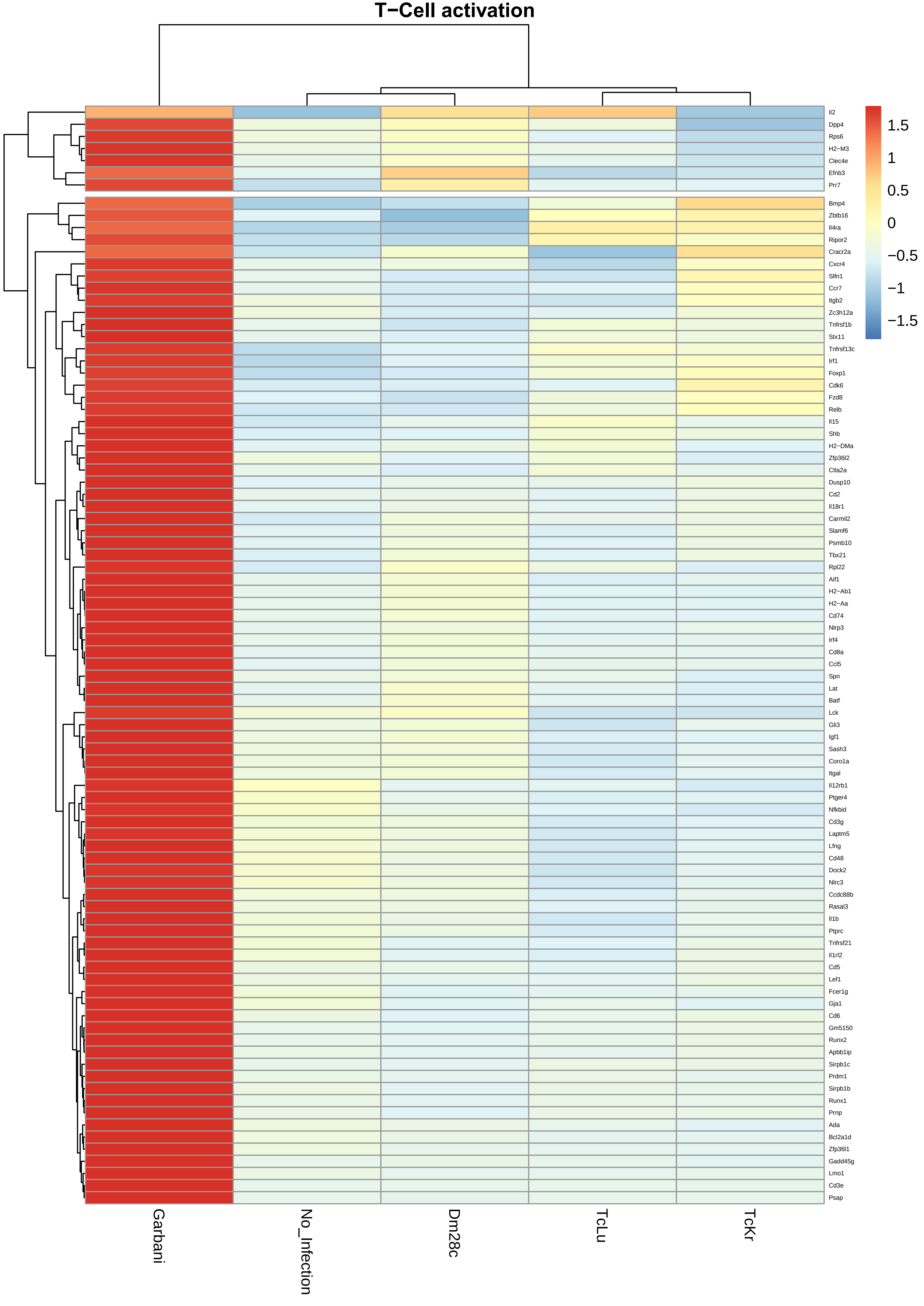
T−Cell activation
Il2
Dpp4
Rps6
H2−M3
Clec4e
Efnb3
Prr7
Bmp4
Zbtb16
Il4ra
Ripor2
Cracr2a
Cxcr4
Slfn1
Ccr7
Itgb2
Zc3h12a
Tnfrsf1b
Stx11
Tnfrsf13c
Irf1
Foxp1
Cdk6
Fzd8
Relb
Il15
Shb
H2−DMa
Zfp36l2
Ctla2a
Dusp10
Cd2
Il18r1
Carmil2
Slamf6
Psmb10
Tbx21
Rpl22
Aif1
H2−Ab1
H2−Aa
Cd74
Nlrp3
Irf4
Cd8a
Ccl5
Spn
Lat
Batf
Lck
Gli3
Igf1
Sash3
Coro1a
Itgal
Il12rb1
Ptger4
Nfkbid
Cd3g
Laptm5
Lfng
Cd48
Dock2
Nlrc3
Ccdc88b
Rasal3
Il1b
Ptprc
Tnfrsf21
Il1rl2
Cd5
Lef1
Fcer1g
Gja1
Cd6
Gm5150
Runx2
Apbb1ip
Sirpb1c
Prdm1
Sirpb1b
Runx1
Prnp
Ada
Bcl2a1d
Zfp36l1
Gadd45g
Lmo1
Cd3e
Psap
1.5
1
0.5
0
−0.5
−1
−1.5
Garbani
No_Infection
Dm28c
TcLu
TcKr

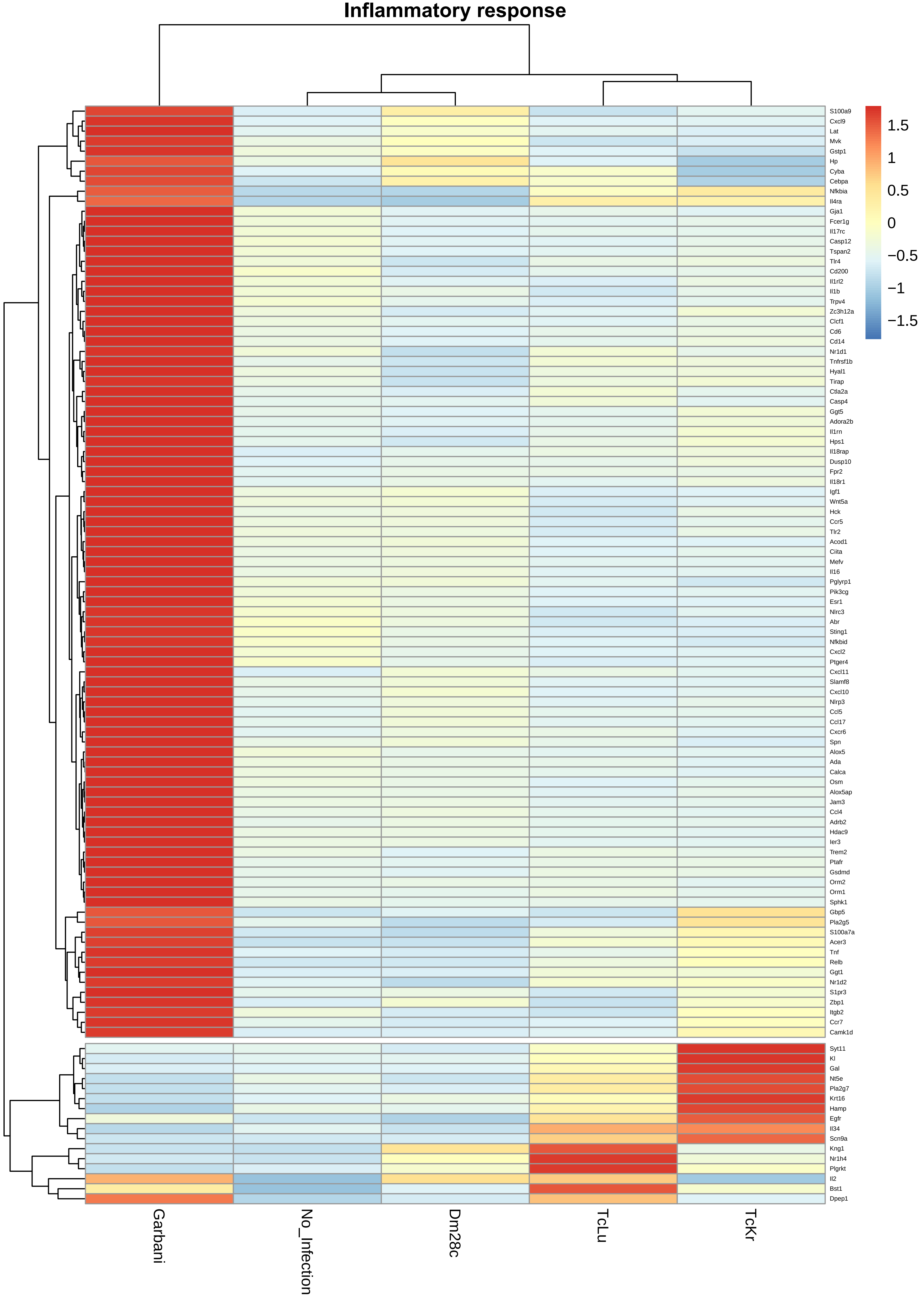

Inflammatory response
S100a9
Cxcl9
Lat
Mvk
Gstp1
Hp
Cyba
Cebpa
Nfkbia
Il4ra
Gja1
Fcer1g
Il17rc
Casp12
Tspan2
Tlr4
Cd200
Il1rl2
Il1b
Trpv4
Zc3h12a
Clcf1
Cd6
Cd14
Nr1d1
Tnfrsf1b
Hyal1
Tirap
Ctla2a
Casp4
Ggt5
Adora2b
Il1rn
Hps1
Il18rap
Dusp10
Fpr2
Il18r1
Igf1
Wnt5a
Hck
Ccr5
Tlr2
Acod1
Ciita
Mefv
Il16
Pglyrp1
Pik3cg
Esr1
Nlrc3
Abr
Sting1
Nfkbid
Cxcl2
Ptger4
Cxcl11
Slamf8
Cxcl10
Nlrp3
Ccl5
Ccl17
Cxcr6
Spn
Alox5
Ada
Calca
Osm
Alox5ap
Jam3
Ccl4
Adrb2
Hdac9
Ier3
Trem2
Ptafr
Gsdmd
Orm2
Orm1
Sphk1
Gbp5
Pla2g5
S100a7a
Acer3
Tnf
Relb
Ggt1
Nr1d2
S1pr3
Zbp1
Itgb2
Ccr7
Camk1d
Syt11
Kl
Gal
Nt5e
Pla2g7
Krt16
Hamp
Egfr
Il34
Scn9a
Kng1
Nr1h4
Plgrkt
Il2
Bst1
Dpep1
1.5
1
0.5
0
−0.5
−1
−1.5
Garbani
No_Infection
Dm28c
TcLu
TcKr

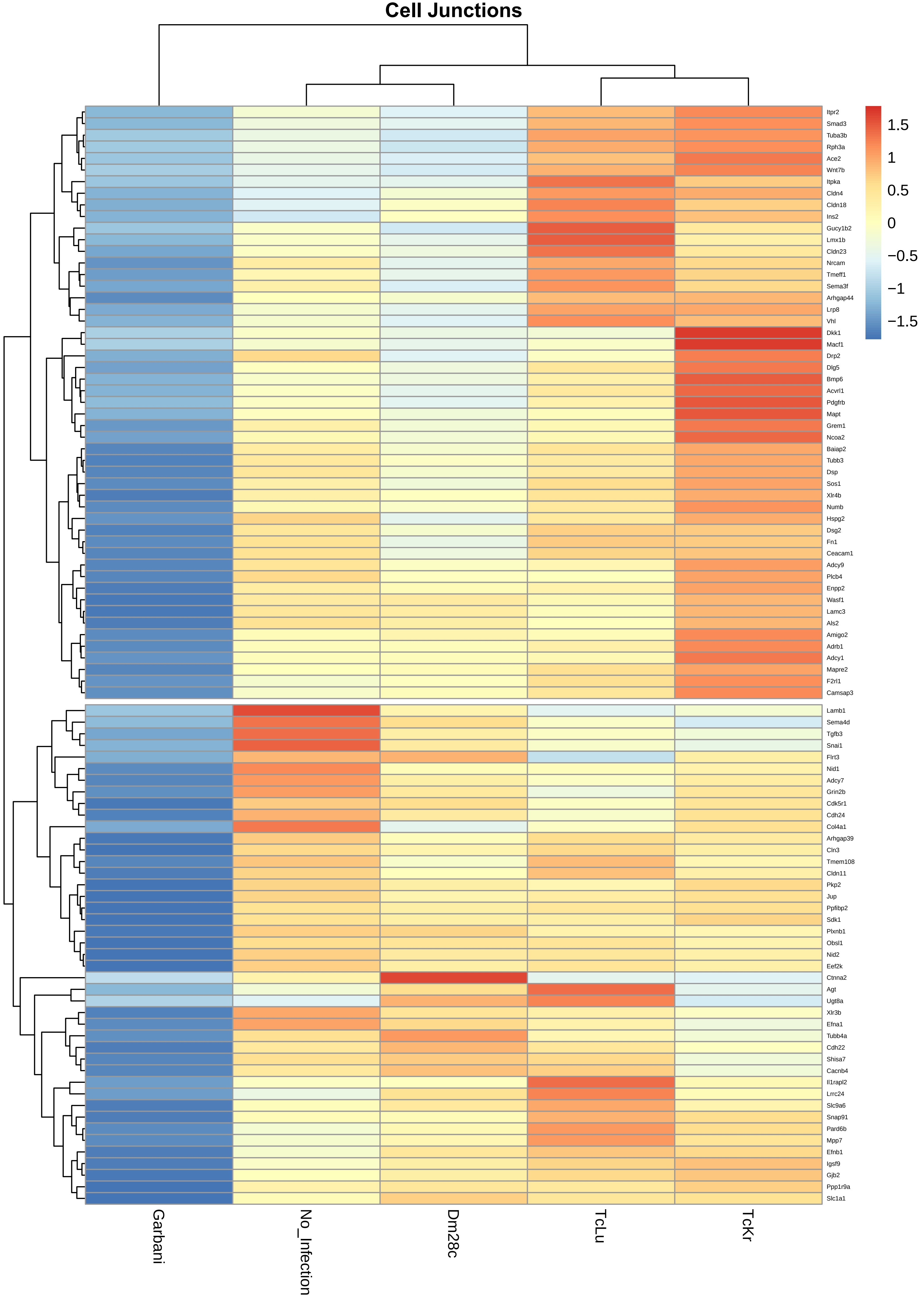
Cell Junctions
1.5
1
0.5
0
−0.5
−1
−1.5
Itpr2
Smad3
Tuba3b
Rph3a
Ace2
Wnt7b
Itpka
Cldn4
Cldn18
Ins2
Gucy1b2
Lmx1b
Cldn23
Nrcam
Tmeff1
Sema3f
Arhgap44
Lrp8
Vhl
Dkk1
Macf1
Drp2
Dlg5
Bmp6
Acvrl1
Pdgfrb
Mapt
Grem1
Ncoa2
Baiap2
Tubb3
Dsp
Sos1
Xlr4b
Numb
Hspg2
Dsg2
Fn1
Ceacam1
Adcy9
Plcb4
Enpp2
Wasf1
Lamc3
Als2
Amigo2
Adrb1
Adcy1
Mapre2
F2rl1
Camsap3
Lamb1
Sema4d
Tgfb3
Snai1
Flrt3
Nid1
Adcy7
Grin2b
Cdk5r1
Cdh24
Col4a1
Arhgap39
Cln3
Tmem108
Cldn11
Pkp2
Jup
Ppfibp2
Sdk1
Plxnb1
Obsl1
Nid2
Eef2k
Ctnna2
Agt
Ugt8a
Xlr3b
Efna1
Tubb4a
Cdh22
Shisa7
Cacnb4
Il1rapl2
Lrrc24
Slc9a6
Snap91
Pard6b
Mpp7
Efnb1
Igsf9
Gjb2
Ppp1r9a
Slc1a1
Garbani
No_Infection
Dm28c
TcLu
TcKr

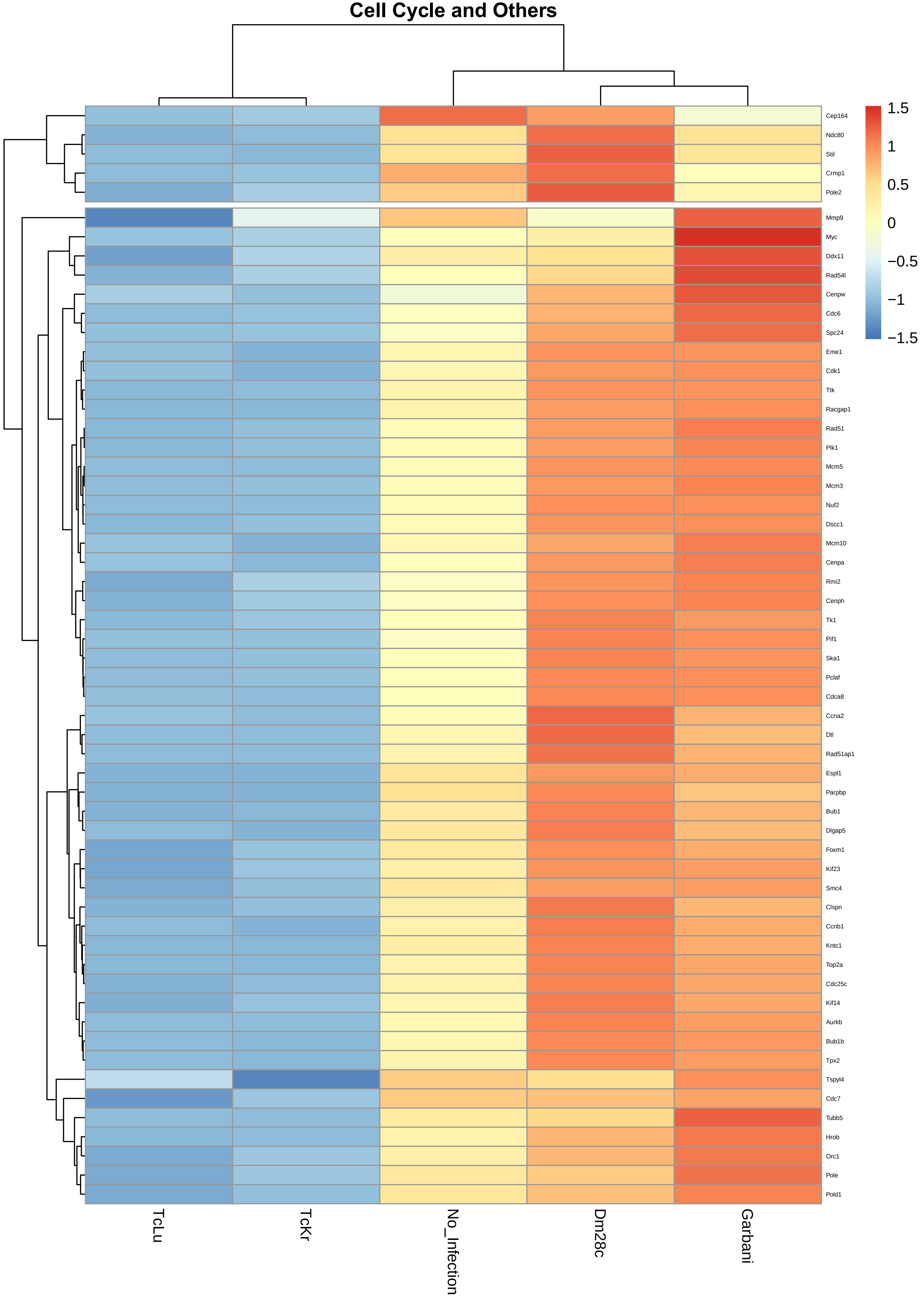
Cell Cycle and Others
1.5
1
0.5
0
−0.5
−1
−1.5
Cep164
Ndc80
Stil
Crmp1
Pole2
Mmp9
Myc
Ddx11
Rad54l
Cenpw
Cdc6
Spc24
Eme1
Cdk1
Ttk
Racgap1
Rad51
Plk1
Mcm5
Mcm3
Nuf2
Dscc1
Mcm10
Cenpa
Rmi2
Cenph
Tk1
Pif1
Ska1
Pclaf
Cdca8
Ccna2
Dtl
Rad51ap1
Espl1
Parpbp
Bub1
Dlgap5
Foxm1
Kif23
Smc4
Clspn
Ccnb1
Kntc1
Top2a
Cdc25c
Kif14
Aurkb
Bub1b
Tpx2
Tspyl4
Cdc7
Tubb5
Hrob
Orc1
Pole
Pold1
TcLu
TcKr
No_Infection
Dm28c
Garbani

Mitotic Cell Cycle Process

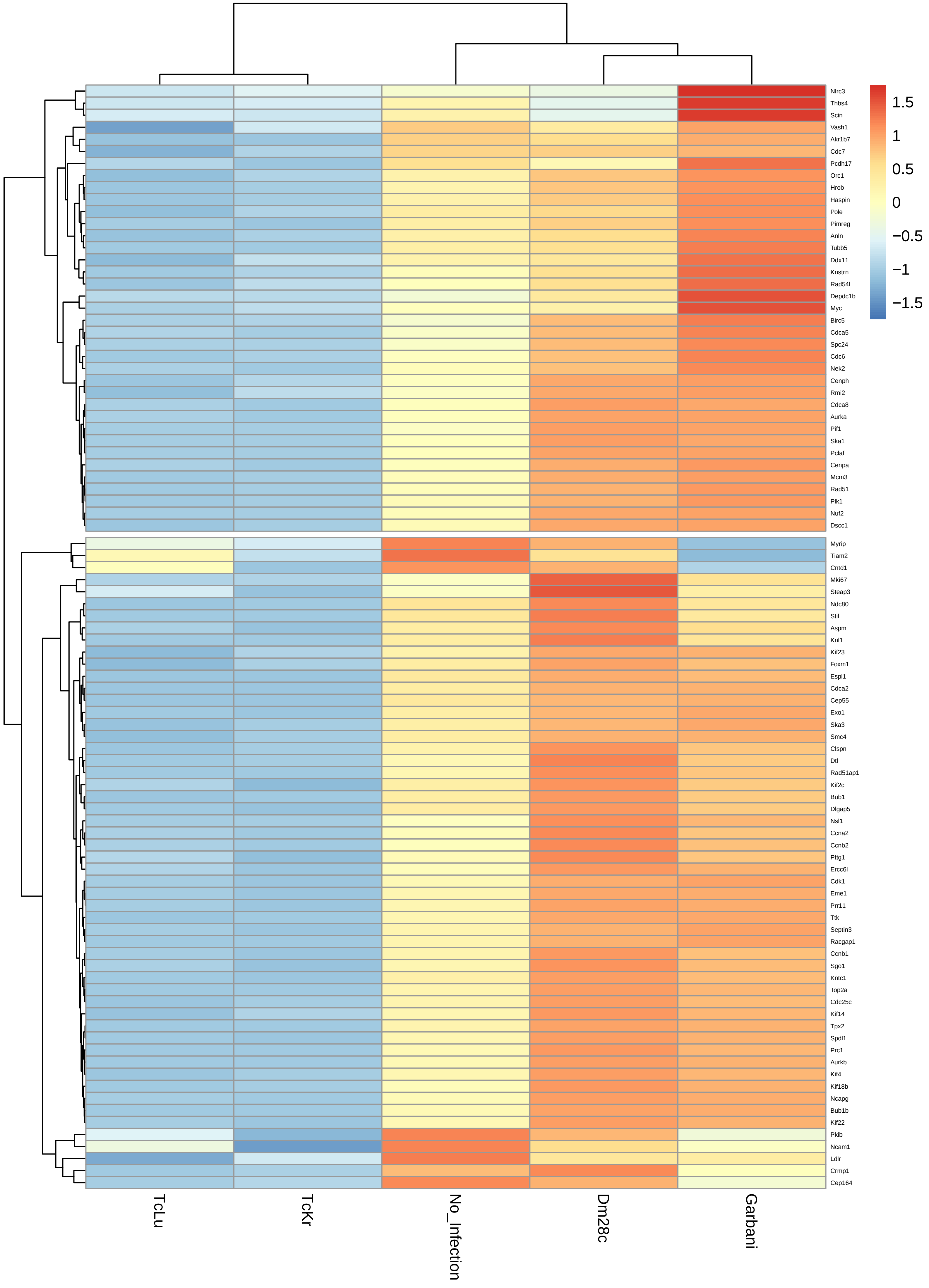

Cell Projections and Morphogenesis

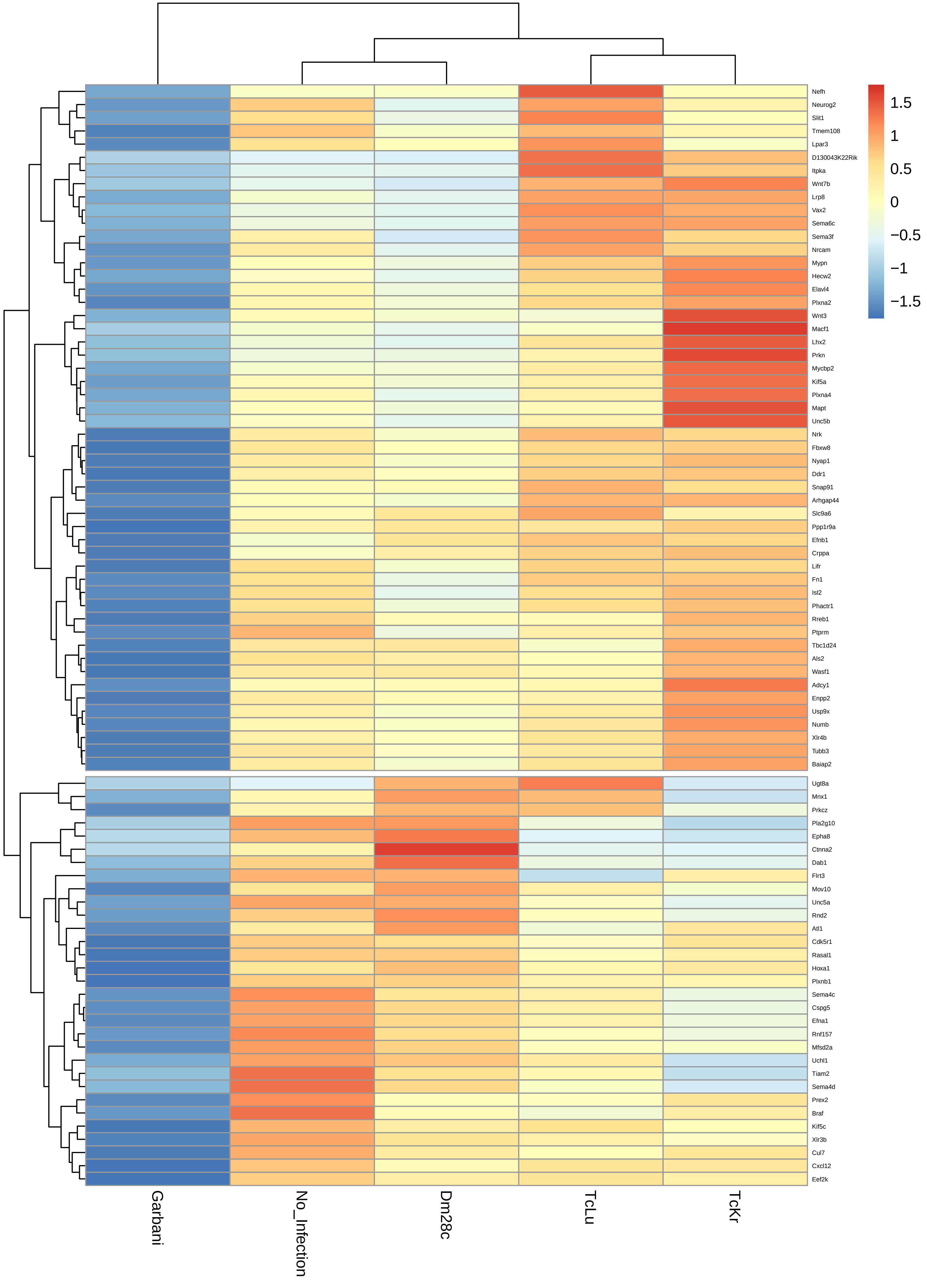

Import to the Cell

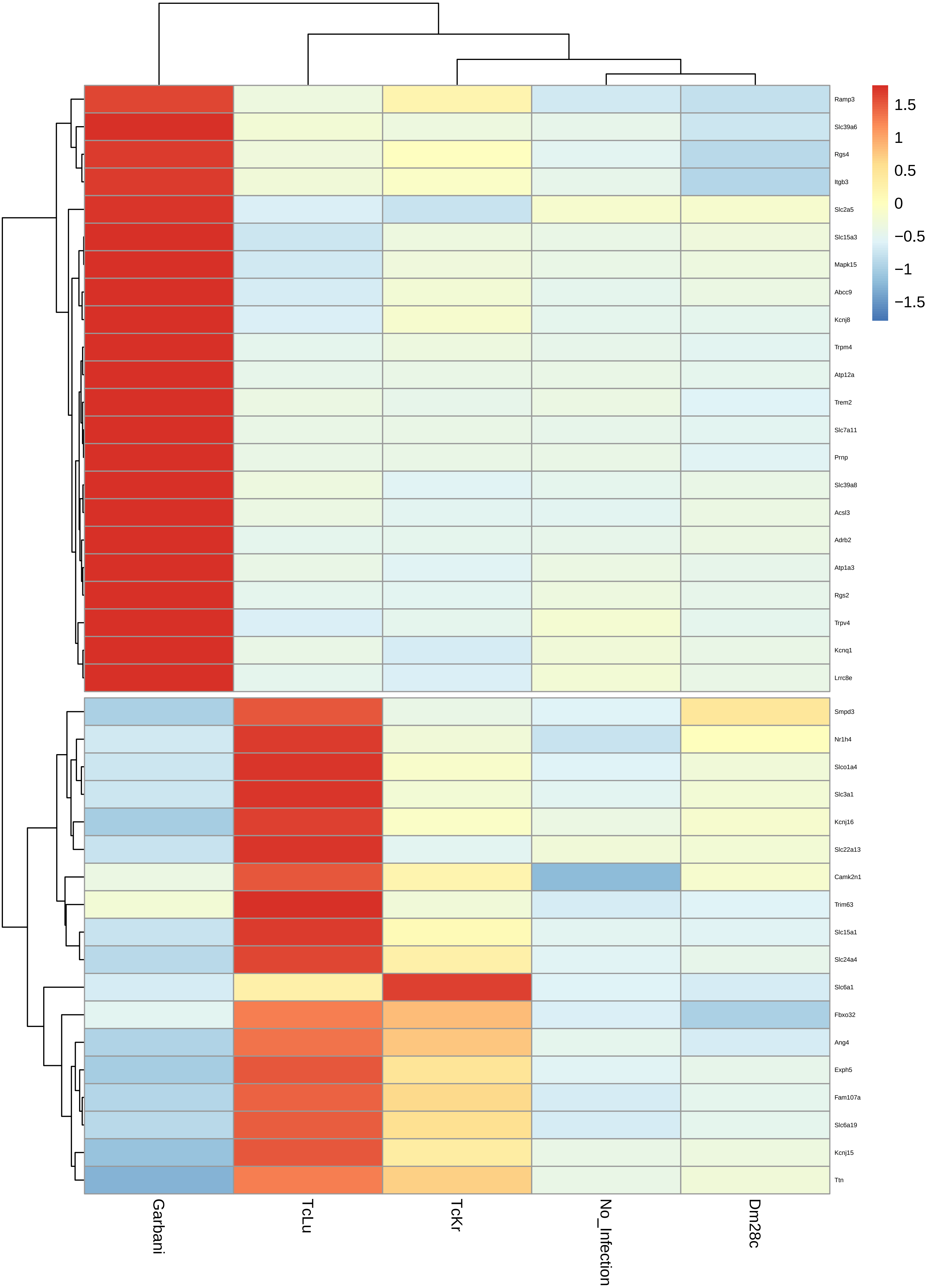

Positive Regulation of Secretion and Import

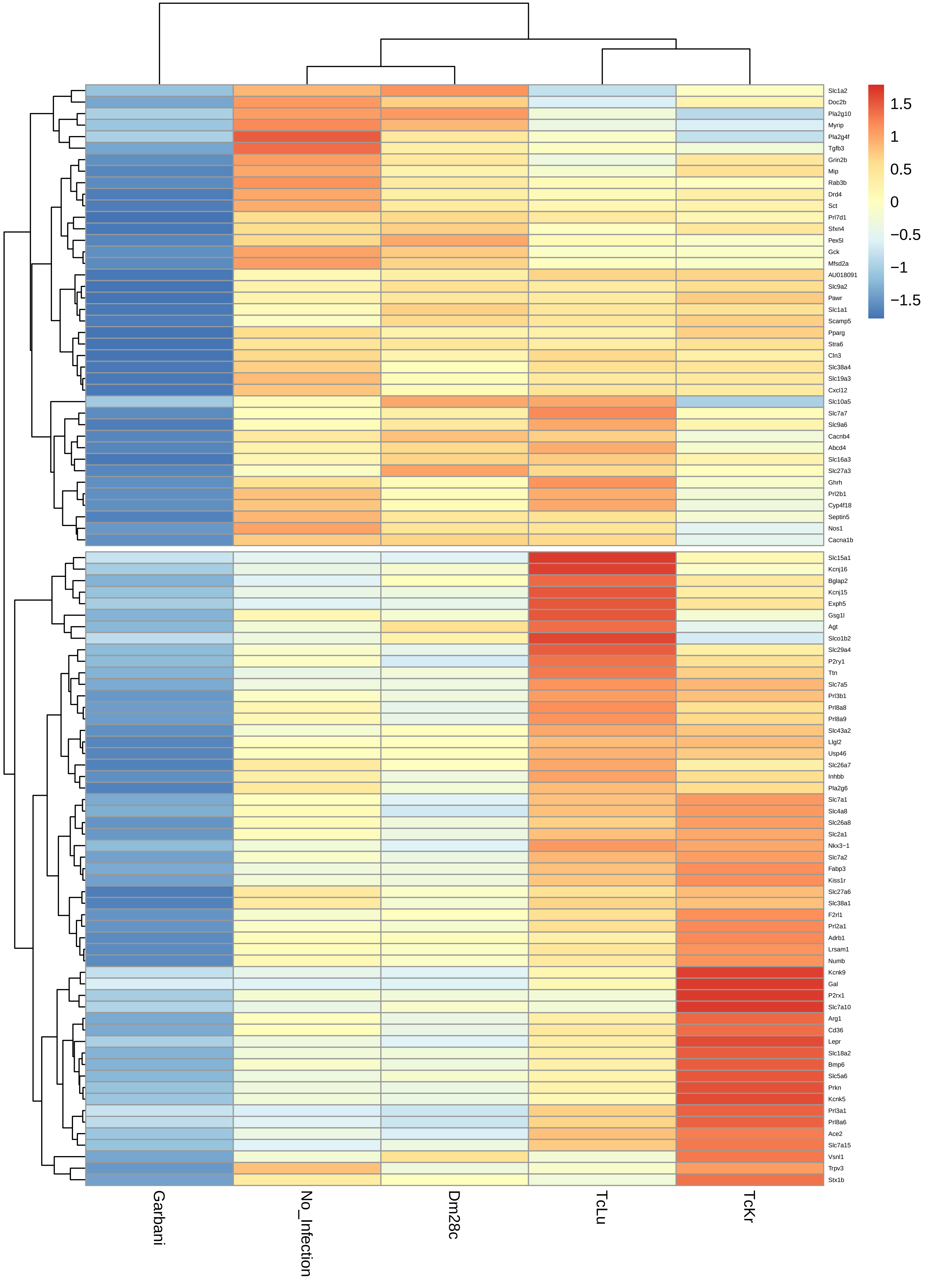

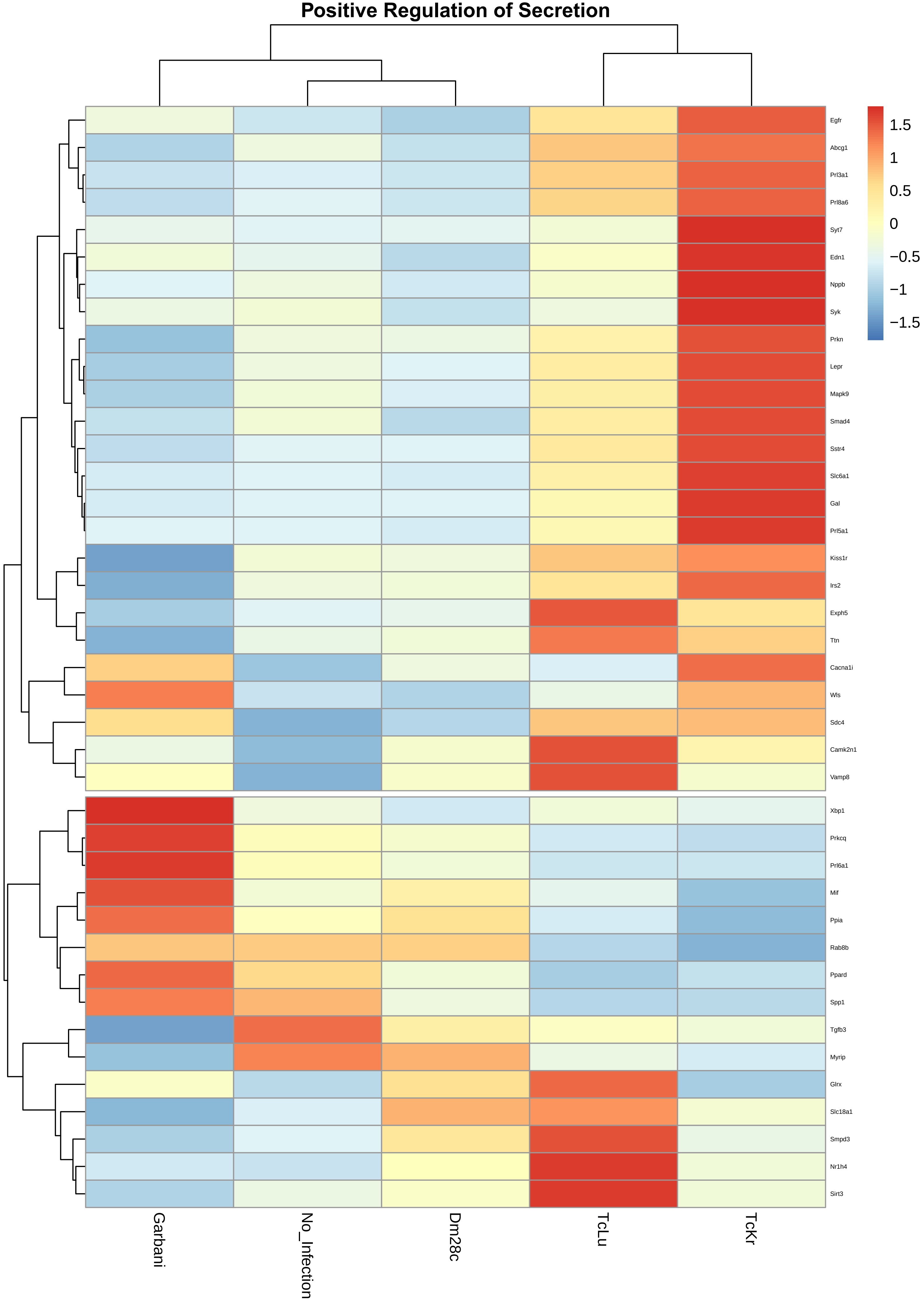

Positive Regulation of Secretion
1.5
1
0.5
0
−0.5
−1
−1.5
Egfr
Abcg1
Prl3a1
Prl8a6
Syt7
Edn1
Nppb
Syk
Prkn
Lepr
Mapk9
Smad4
Sstr4
Slc6a1
Gal
Prl5a1
Kiss1r
Irs2
Exph5
Ttn
Cacna1i
Wls
Sdc4
Camk2n1
Vamp8
Xbp1
Prkcq
Prl6a1
Mif
Ppia
Rab8b
Ppard
Spp1
Tgfb3
Myrip
Glrx
Slc18a1
Smpd3
Nr1h4
Sirt3
Garbani
No_Infection
Dm28c
TcLu
TcKr

Ribosomal Citoplasmatic proteins

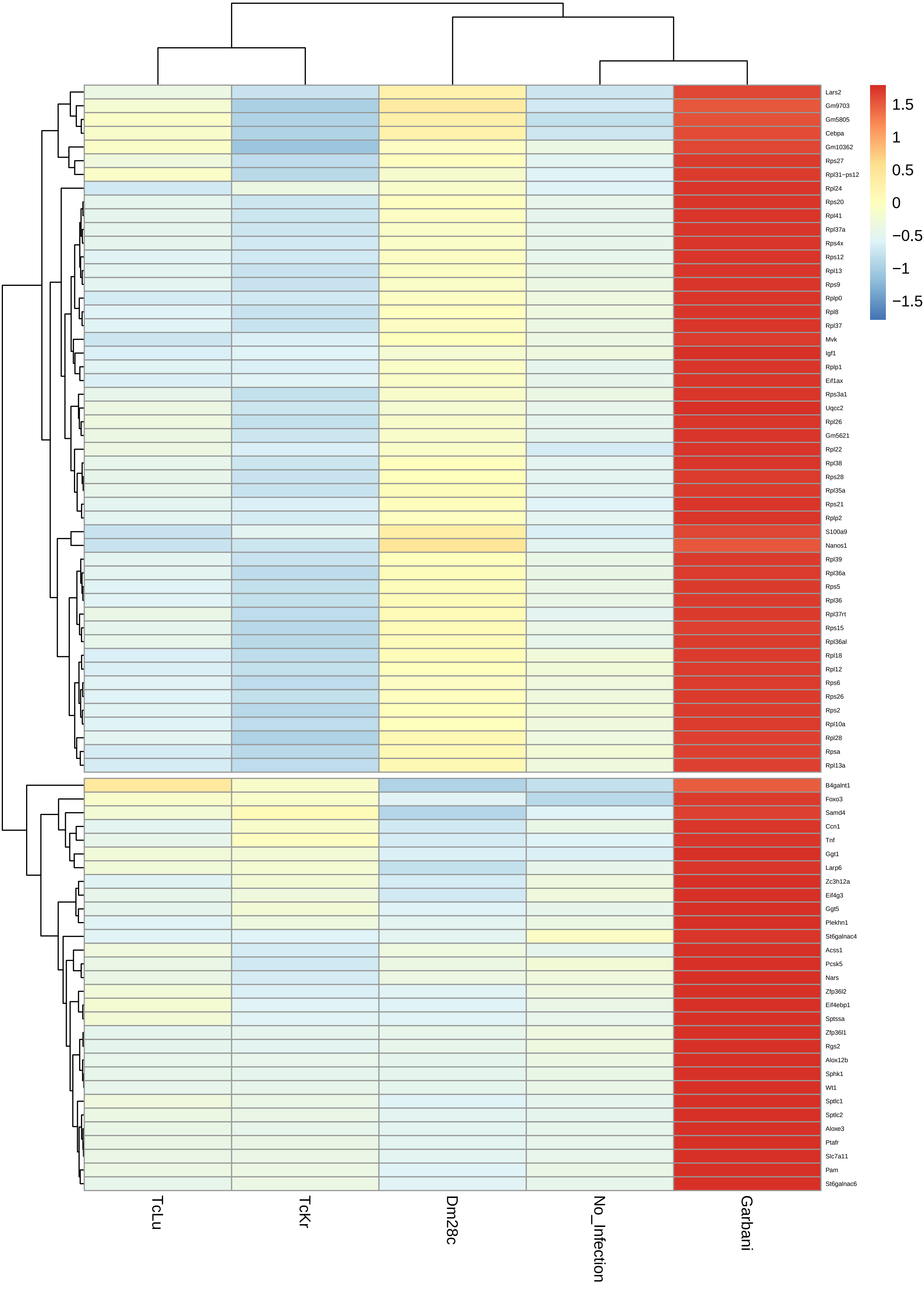

Supplement: Supplemental file 1 — Fig. S1 to S4. Download spectrum.02504-22-s0001.pdf, PDF file, 3.6 MB [file spectrum.02504-22-s0001.pdf]
